# Exploring the Functionality of the Krüppel-like Factors in Kidney Development, Metabolism, and Diseases

**DOI:** 10.3390/life14121671

**Published:** 2024-12-17

**Authors:** Itzel S. Salmon-Cabrales, David A. de la Garza-Kalife, Gabriel García-González, Ana E. Estrada-Rodríguez, Marco Antonio Jiménez-Gutiérrez, Michelle G. Santoyo-Suárez, Oscar Rodríguez-Núñez, Elsa N. Garza-Treviño, Diego F. Benítez-Chao, Gerardo R. Padilla-Rivas, Jose Francisco Islas

**Affiliations:** 1 Laboratorio de Terapia Celular, Departamento de Bioquímica y Medicina Molecular, Facultad de Medicina, Universidad Autónoma de Nuevo León, Av. Dr. José Eleuterio González 235, Monterrey 64460, Nuevo León, Mexico; itzel.salmonc@uanl.edu.mx (I.S.S.-C.); david.delagarzaka@uanl.edu.mx (D.A.d.l.G.-K.); gabriel.garciagnzl@uanl.edu.mx (G.G.-G.); marcojimenez9999@gmail.com (M.A.J.-G.); s.antoyo@hotmail.es (M.G.S.-S.); oscar.rodrigueznu@uanl.edu.mx (O.R.-N.); elsa.garzatr@uanl.edu.mx (E.N.G.-T.); diego.benitezch@uanl.edu.mx (D.F.B.-C.); gerardo.padillarv@uanl.edu.mx (G.R.P.-R.); 2 Departmento de Ciencias Básicas, Vicerrectoría de Ciencias de la Salud, Universidad de Monterrey, Ignacio Morones Prieto 4500, Jesus M. Garza, San Pedro Garza García 66238, Nuevo León, Mexico; ana.estradar@udem.edu

**Keywords:** Krüppel-like factors, kidney, development, metabolism, disease, regulation

## Abstract

The kidneys contribute to the overall health of an organism by maintaining systemic homeostasis. This process involves various biological mechanisms, in which the Krüppel-like factors (KLFs), a family of transcription factors, are essential for regulating development, differentiation, proliferation, and cellular apoptosis. They also play a role in the metabolic regulation of essential nutrients, such as glucose and lipids. The dysregulation of these transcription factors is associated with the development of various pathologies, which can ultimately lead to renal fibrosis, severely compromising kidney function. In this context, the present article provides a comprehensive review of the existing literature, offering an enriching analysis of the findings related to the role of KLFs in nephrology, while also highlighting their potential therapeutic role in the treatment of renal diseases.

## 1. Introduction

The kidneys are intricate organs that are vital in maintaining the body’s electrolyte, acid–base, and waste balance [1] by filtering and reabsorbing substances and eliminating toxins from the bloodstream [2]. Additionally, the kidneys perform endocrine functions, such as hormone production, e.g., erythropoietin, 1-α hydroxylase, and renin, which are involved in erythropoiesis, calcitriol synthesis, and blood pressure regulation, respectively [3]. It is important to note that to maintain their proper cellular function, particular responses are determined by effectors, such as transcription factors, which respond to external signals resulting in the modulation of gene expression, thereby tightly controlling physiological functions [4]. Amongst the responses of the different transcription factors, in recent years, the Krüppel-like factor (KLF) family of transcription factors has been the object much attention for its role in the development and functional maintenance of the kidneys [5].

Briefly, the KLF family is a 17-member family of transcription factors characterized by three zinc fingers (Cys2/His2) with highly conserved C-terminal domains [6]. Regarding renal function, KLFs have been associated with several biological processes, including differentiation, terminal maturation, and the maintenance of the structure and function of the glomerular filtration barrier (GFB), as well as the protection of glomerular endothelial cells (ECs) and podocytes from inflammatory damage and the development of fibrosis [7]. Its members, such as KLF5, have been associated with the promotion of inflammation and the development of tubulointerstitial fibrosis by positively regulating pro-inflammatory cytokines [8,9] and cell proliferation by activating the pathways that induce cell growth [10]. Structural variations in their regulatory domains determine the variety of roles that the KLFs play in renal biological processes. Through their interactions with various coactivators and/or corepressors, their domains enable them to activate or repress the promoter activity of their target genes [11,12]. The dysregulation of the expression of the KLFs can affect the physiological functions in which they are involved, promoting the development of different diseases [13,14].

This article aims to provide an overview, based on a comprehensive review, of the most relevant findings related to the involvement of KLFs in the various biological processes of the kidneys. We emphasize the importance of the KLFs during renal development, their role in regulating the metabolic processes of essential macronutrients, such as glucose and lipids, and their implications in the onset and progression of significant renal diseases, such as acute kidney injury (AKI), chronic kidney disease (CKD), and diabetic kidney disease (DKD), highlighting KLFs that exhibit renal-protective effects.

## 2. Krüppel-like Factors

Krüppel-like factors make up a family of zinc-finger-motif DNA-binding transcription regulators. Their name derives from the initial discovery of the Krüppel protein in *Drosophila melanogaster* in 1993, a protein that participates in regulating fly embryo segmentation [15], in a study on non-bilateral lineages that included 48 species from the Eukarya kingdom. The data presented are relevant, as they were used to trace the evolutionary history of this family of transcription factors, determining that the KLF genes originated in the opisthokont stem lineage and the specificity protein (SP) genes in metazoans, excluding ctenophores [16]. In humans, the KLFs consist of 17 members, which, as mentioned, exhibit three highly conserved Cys2His2-type zinc finger motifs in their carboxyl-terminal regions. Each zinc finger is formed by two cysteine residues and two histidine residues that coordinate a zinc ion. Typically, the amino acid sequence that forms the zinc finger motif is CX(2-4)-CX(12)-HX(3-5)-H, which maintains < ns a fixed length of 23 or 21 amino acids for their motifs [17]. The evolutionarily conserved, DNA-binding domain structure shows structural homology with SP, which has led to the consideration of KLFs as a subgroup of the SP/KLF family [18]. These motifs facilitate binding to both the GC-rich proximal promoter regions and the CACCC elements (GT boxes) within the promoter regions of multiple genes [19]. The zinc finger motifs can bind to DNA through a specific recognition code, determined by the non-conserved amino acid residues in their alpha-helical region. The positions of the amino acid residues in the zinc fingers that interact with the DNA bases wrap around the DNA and connect with three nucleotides [20].

**Figure 1:**
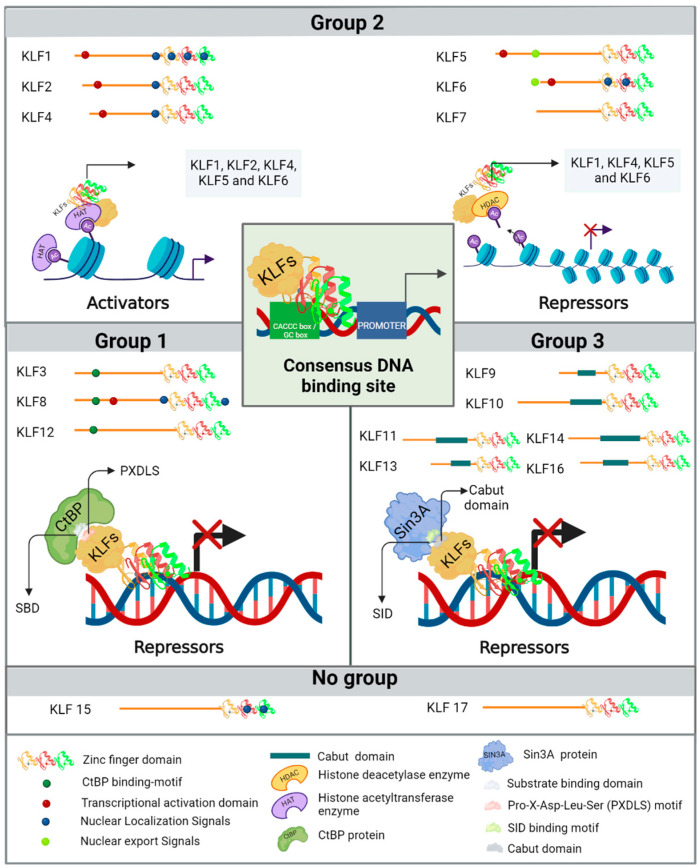
Different groups of KLFs based on their phylogenetic and structural characteristics of the amino-terminal region. (Group 1) includes KLF3, KLF8, and KLF12, whose primary function is transcriptional repression through interaction with the CtBP. (Group 2) comprises KLF1, KLF2, KLF4, KLF5, KLF6, and KLF7; these KLFs exhibit diverse activities and can function as either transcriptional activators or repressors. Finally, (Group 3) includes KLF9, KLF10, KLF11, KLF13, KLF14, and KLF16, which exert repressive effects similar to those of Group 1, but their action depends on their interaction with the transcriptional corepressor Sin3A. KLF15 and KLF17 are not assigned to a specific group because their protein interaction mechanisms are not yet fully explained, and they are also phylogenetically more distantly related.

The phylogenetic features that mark the evolutionary distance amongst the KLF family members, coupled with the specific structural traits in their less conserved amino-terminal regions, have resulted in the division of the KLFs into three distinct groups [21]. The first group contains KLF3, KLF8, and KLF12, which harbor a Pro-X-Asp-Leu-Ser (PXDLS) motif, where X represents a hydrophobic amino acid. This motif facilitates interaction with the amino-terminal substrate-binding domain (SBD) of the C-terminal binding proteins (CtBP), aiming to repress transcription [11,22,23,24]. The second group includes KLF1, KLF2, KLF4, KLF5, KLF6, and KLF7, which, along with KLF8 from group 1 and KLF15 (the latter by homology), and except for KLF7, possess a transcriptional activation domain (TAD) in their N-terminal regions [11,23]. KLF1–2, KLF4–6, and KLF13 (the latter from group 3) can, in addition to binding to consensus sites in DNA, interact with histone acetyltransferase enzymes, such as cAMP response element-binding protein (CREB) binding protein (CBP), p300, and p300/CBP-associated factor, facilitating chromatin remodeling and promoting transcriptional activity in DNA regions regulated by KLFs [11,23,25,26]. The interaction of KLFs with epigenome-modifying enzymes can occur in different ways, such as when they are acetylated by histone acetyltransferases, promoting the acetylation of genes and inducing their expression, or conversely when KLFs bind to specific promoters to recruit histone deacetylase enzymes and locally promote the deacetylation of histone proteins, repressing gene expression. An example of this is how KLF4 can recruit p300 and acetylate itself, promoting the activation of genes such as the intestinal alkaline phosphatase gene in LS174T colon cancer cells, or repress gene expression by deacetylating p53 through the recruitment of HDAC3 to its promoter region [27]. The binding of KLF1, KLF4, KLF5, and KLF11 (the latter from group 3) to histone deacetylase enzymes suppresses their transcriptional activity. Therefore, this second group of KLFs can modulate both the activation and repression of gene expression at the transcriptional level, depending on the biological context and the gene regulatory region through which they are operating [19]. Lastly, in the third group, members KLF9, KLF10, KLF11, KLF13, KLF14, and KLF16 contain a Cabut domain in their N-terminal section that encompasses a Sin3 interaction domain (SID), which explains their activities as transcriptional repressors [7]. KLF15 and KLF17 are not classified within these three phylogenetic groups because their protein interaction domains have not yet been determined. Finally, researchers have determined that various KLFs also contain nuclear localization signals (NLS) and nuclear export signals (NES) which regulate their subcellular localization, as illustrated in Figure 1.

## 3. KLFs in Kidney Physiology

The kidneys play essential roles in maintaining homeostasis in the body, mediated by hormonal signaling processes and their interaction with transcription factors [28,29]. One family of transcription factors involved in regulating renal function is the KLFs [28,30,31], which are expressed in various parts of the nephron, contributing to both its structure and cellular composition [32].

These aspects are fundamental in the cellular biology of kidneys, and understanding them provides better insights into the role of KLFs in them, clarifying their influence on determining cellular function and behavior.

The kidneys are divided into two specific regions: the renal cortex and the renal medulla. These two regions make up the renal parenchyma, which is the functional tissue of the kidney. The functional and structural unit of the kidney is the nephron, composed of the glomerulus and the tubular system [1,33].

Surrounded by a cup-shaped structure called Bowman’s capsule, the glomerulus is where blood filtration occurs. The parietal epithelial layer is composed of parietal cells that play a structural maintenance role [1], and the visceral layer is formed by podocytes, which are perivascular cells enveloping the outer layer of the basal membrane of the glomerular capillaries (GCs), together forming the GFB [34,35]. The integrity of this barrier is maintained by the surface proteins expressed by ECs, the selective adhesion molecules of ECs that mediate cell-to-cell adhesion and vascular permeability [35,36], and the slit diaphragms (SD) between the foot processes of podocytes, ensuring proper glomerular filtration [37,38,39]. KLF4 collaborates with histone deacetylases (HDACs), specifically, HDAC1 and HDAC3, to upregulate the expression of E-cadherin, podocin, and nephrin. E-cadherin is an adhesion molecule, while podocin and nephrin are crucial for the formation and maintenance of the SD, a specialized adhesion structure in podocytes [36,37,38]. KLF4 also induces the expression of cytokeratin 8 (K8) and K18, which, like podocins, participate in cytoskeletal organization [36]. Together, the expression of these proteins maintains the structure and functionality of podocytes by modulating cellular adhesion and polarity.

Maintaining cell adhesion and polarity is crucial to prevent acquiring mesenchymal characteristics. KLF4 is also involved in downregulating mesenchymal markers, such as vimentin and α-smooth muscle actin (α-SMA), in podocytes [36]. In addition to being expressed under normal conditions in podocytes, vimentin is also expressed in mesenchymal cells, which generally lack intercellular adhesion and polarity, providing them with resistance to migration-related stress [39,40]. In contrast, α-SMA contributes to the motility and contraction of the cytoskeleton [41], allowing greater cell mobility and the ability to migrate to sites of injury or inflammation. Therefore, KLF4 prevents structural damage and the progression of diseases by inhibiting epithelial-to-mesenchymal transition (EMT). Moreover, KLF6 has been associated with maintaining mitochondrial function and preventing cell death in podocytes. This is due to KLF6’s ability to bind to the promoter region of the cytochrome c oxidase assembly gene (SCO2) and positively regulate it. Continuous expression of SCO2, a metallochaperone, is crucial for transporting copper ions to electron carriers in the mitochondrial electron transport chain. This process, including cytochrome c, is vital to prevent the activation of the intrinsic apoptotic pathway in podocytes. By doing so, it averts glomerular damage and maintains its filtration capability [31].

Regarding energy metabolism, SCO2 can modulate the metabolic switch from glycolysis to oxidative phosphorylation (OXPHOS) in hematopoietic stem cells with Fanconi anemia as a compensatory mechanism in affected cells to ensure functional energy metabolism. This could suggest that the proper maintenance of OXPHOS is mediated partly by SCO2 in renal cells that rely on fatty acid oxidation to meet their energy needs [42].

Fenestrated ECs possessing transcellular pores of 60 to 100 nm confer GC selective permeability for molecules according to their size and charge [1,43]. KLF2 contributes to regulating the size and distribution of these pores, preventing uncontrolled solute permeability by inhibiting the phosphorylation of the myosin light chain, thus avoiding the contraction of the cytoskeleton that would reduce the size of the ECs and lead to the formation of gaps between them [44]. Another way to maintain proper glomerular filtration is through the modulation of angiogenesis mediated by VEGF-A, the expression of which can be downregulated by KLF2. This prevents the occurrence of an excess of unnecessary or dysfunctional blood vessels that could disrupt hemodynamic balance and affect blood pressure [45].

The role of KLF4 in ECs relates to the reduction of inflammation through the downregulation of adhesion molecules, such as VCAM1, induced by TNF-α. This is achieved by inhibiting the expression of the p65 subunit of nuclear factor kB (NF-κB), which is necessary for the activation of this transcription factor and its binding to the VCAM1 promoter. In this way, KLF4 modulates the adhesion and recruitment of lymphocytes to ECs, preventing chronic inflammation [46]. Moreover, the anti-inflammatory effect of KLF4 in ECs has been shown to improve the condition of patients with vascular lesions due to ischemic stroke by regulating the endothelial expression of other inflammatory cell adhesion molecules such as E-selectin, intercellular adhesion molecule 1 (ICAM-1), as well as VCAM-1, NF-kB, and tight junction proteins [47]. Mesangial cells are part of the mesenchymal stromal cell group, along with fibroblasts, pericytes, and vascular smooth muscle cells (VSMCs) [48]. As stromal cells, they organize the structure of the glomerulus and contribute to the homeostasis of ECs and podocytes by directing the immune response and repair after glomerular injury [48,49]. Mesangial cells support GCs and the mesangial cells extending into the extraglomerular mesangium, which are part of the juxtaglomerular apparatus, contributing to the regulation of blood pressure and fluid volume by secreting renin [50].

In a study using C57BL/KsJ db/db mice with overexpressed KLF4 via a lentiviral vector in podocytes, researchers demonstrated, through histological staining with periodic acid-Schiff, that renal injury was mitigated by attenuating the expansion of the mesangial matrix and the proliferation of mesangial cells in the glomerulus. It is worth mentioning that a more thorough analysis of these results was not presented. Therefore, the question of how the overexpression of KLF4 in podocytes appears to affect both the proliferation of mesangial cells and the production of mesangial matrix remains unanswered.

This event suggests the prevention of the overproduction of type IV and V collagens and fibronectin by mesangial cells, thereby preventing glomerular fibrosis and the development of fibrotic kidney diseases [51,52].

Between the parietal layer and the visceral layer of Bowman’s capsule, a urinary space known as the “Bowman’s space” is formed. This space represents the beginning of the urinary system and is contiguous with the proximal convoluted tubule (PCT) in the renal cortex [53]. The PCT, which is the first segment of the nephron’s tubular system, receives glomerular ultrafiltrate through epithelial cells (EpC), reabsorbing glucose, amino acids, and minerals such as phosphate, chloride, and bicarbonate, as well as secreting hydrogen ions and toxins produced by cellular metabolism and xenobiotics present in the filtrate [54]. The expression of KLF4 and KLF11 mitigates inflammation and fibrosis by decreasing the expression of cytokines MCP-1, MIP-3α, and IL-8, induced by TGF-β1. This is possibly related to the phosphorylation of KLF4 that induces the SMAD and p38/MAPK signaling pathways in VSMCs [55] or through binding to p65 to inhibit NF-kB signaling. A decrease of these inflammatory cytokines limits the production of type I collagen and tissue fibrosis [56].

In EpC, KLF15 can decrease the expression of these fibrogenic components by negatively regulating the MAPK pathways, which, when activated, contribute to the production of TGF-β1 and other profibrogenic factors. Thus, KLF15 also modulates the fibrogenic response and helps prevent the accumulation of extracellular matrix (ECM) [57].

The specific expression of genes within each segment of the renal tubule determines its respective role [32]; unfortunately, the expression and function of KLFs in the subsequent segments of the nephron’s tubular system remain incompletely understood. Following the PCT, the loop of Henle consists of a thin descending limb, a thin ascending limb, and a thick ascending limb; it contributes to the regulation of urine concentration by passively reabsorbing water into the medullary interstitium. In the thin descending limb, 15% of water and potassium are reabsorbed, while sodium chloride (NaCl) is only modestly recovered. In contrast, in both the thin ascending limb and the thick ascending limb, approximately 25% of NaCl is reabsorbed. Urea reabsorption in the ascending limb of the loop of Henle increases solute concentration in the extracellular space. At the same time, its excretion is determined by glomerular filtration and tubular reabsorption, allowing it to play an important role in promoting concentrated urine production and maintaining water balance [58]. The distal convoluted tubule (DCT) performs the functions of reabsorbing sodium, potassium, and chloride, as well as secreting hydrogen and potassium ions. Finally, the collecting tubule, which reabsorbs the same electrolytes as the DCT, drains into the renal papillae [1,53].

**Figure 2:**
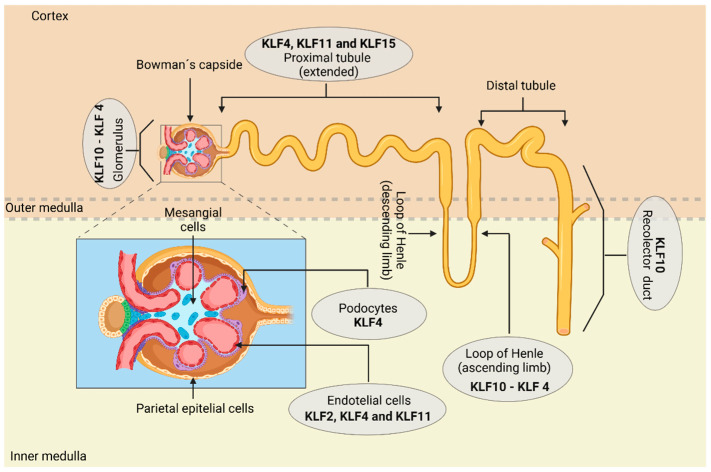
The basic structure of the nephron consists of a renal corpuscle and a renal tubule. The renal tubule is divided into several segments: the proximal tubule, where early solute reabsorption occurs to prevent accumulation and nephrotoxicity; the loop of Henle; the DCT; and finally, the collecting tubule, where urine is concentrated through coordinated processes of reabsorption and secretion. Knowledge of the location of the main cellular groups within the nephron is crucial to accurately identify the specific gene expression of KLFs. It has been shown that KLF10 is the most prevalent transcription factor in the glomerulus, inner medulla, thin ascending limb of the loop of Henle, and inner medullary collecting duct (IMCD). KLF4 follows KLF10, being detected in large quantities in both the glomerulus and loop of Henle. Additionally, KLF2, KLF4, and KLF11 are expressed in renal ECs.

Therefore, the main function of KLFs in the kidneys can be summarized as preserving and regulating cell adhesion, structure, ECM production, the GFB, and inflammation of the cell lineage that composes it. This enables the modulation of glomerular filtration, secretion, and elimination of unnecessary toxins from the body. Figure 2 illustrates the basic structure of the nephron and highlights the locations of its main cell groups, integrating the specific gene expression of KLFs within them. Table 1 summarizes the specific functions of each KLF involved in the kidney’s functional regulation processes.

### 3.1. Klf in Kidney Development

The process of organogenesis involves the division and organization of cells to establish the foundational structures of the embryo. Cell cycle arrest plays a crucial role in this process, as it enables the progression toward terminal differentiation, a vital step in acquiring specialized functions [59].

The intricate progression of the cell cycle and cellular differentiation at a molecular level is regulated by signaling pathways and transcription factors [60]. This principle highlights the importance of the KLF family in kidney development. In embryonic cell cycle regulation, in vitro studies suggest that KLF5 promotes podocyte survival by blocking the MAPK ERK/p38 pathway and by decreasing the expression of apoptosis-related proteins such as Bax, caspase-3, caspase-8, and caspase-9, while increasing the expression of the antiapoptotic protein Bcl-2, thus inhibiting cell cycle arrest and apoptosis [61,62,63]. By contrast, in diabetic nephropathy patient serum or in a model of HK2 cells with nephritis induced by high glucose concentrations, researchers demonstrated that the inhibition of KLF5 through miR-214-5p protects against tubular injury under these physiological conditions by inhibiting apoptosis and restoring cell proliferation. Additionally, protein levels of inflammatory factors such as IL-6, TNF-α, and IL-1β are reduced, along with the production of reactive oxygen species (ROS) and the malondialdehyde content [64]. A possible mechanism by which the inhibition of KLF5 promotes apoptosis has even been demonstrated in prostate cancer cells, where this inhibition, along with the proteins Stat5a/b and ICAM-1 through bioactive plant-derived agents, has been associated with the positive regulation of the Bax/Bcl-2 ratio. This relationship regulates apoptosis; specifically, the inhibition of KLF5 induces an increase in the expression of Bax, which translocates to the mitochondria, promoting the release of cytochrome c and consequently the activation of caspase-3 and PARP, key proteins in the apoptotic process. The expression of Bcl-2 is not significantly affected during KLF5 downregulation [65].

**Figure 3:**
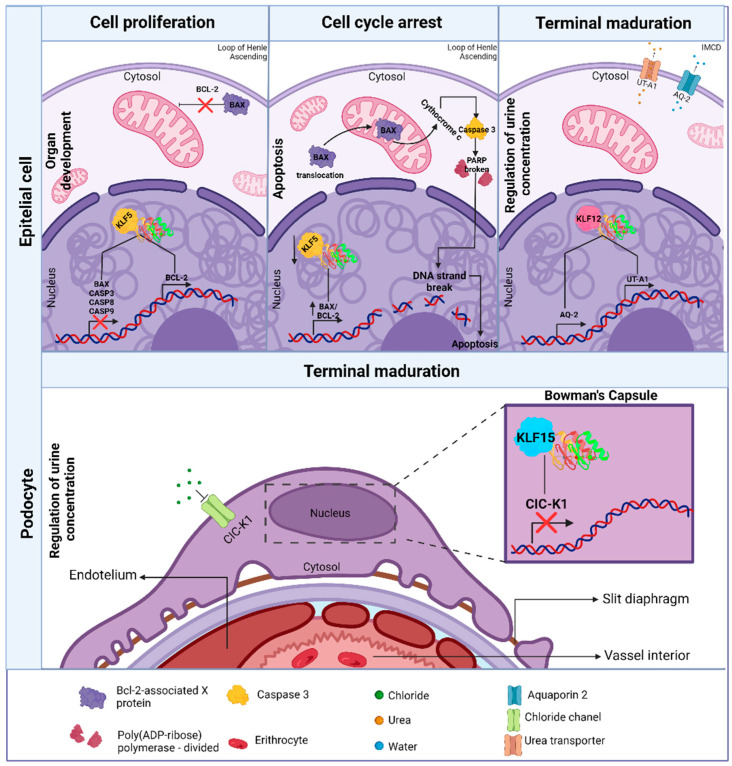
KLF involvement in regulating renal organogenesis and the postnatal maturation of nephrons. The KLFs promote the survival of EpC and podocytes. Interestingly, the survival of podocytes mediated by KLF5, through the inhibition of the MAPK pathway, may seem like a contradictory description, as ERK has been shown to positively induce the expression of anti-apoptotic proteins, such as Bcl-2. ERK is typically associated with promoting cellular survival; however, its interaction with p38 can lead to apoptosis under severe stress conditions. For this reason, KLF5 may influence the balance between the activation of ERK and p38, so that, by modulating this signaling, cellular survival is favored. They also participate in the postnatal maturation of nephrons, the development of electrolyte balance, and proper urine concentration.

The complete maturation of the nephrons occurs postnatally and is essential for the kidneys to develop their maximum urinary concentrating ability. Various mechanisms are outlined below to illustrate how KLF12 and KLF15 are fundamental in this process. KLF15 acts as a negative regulator of the chloride channel ClC-K1, which is expressed in the EpC thin ascending limb of the loop of Henle during postnatal development. This KLF15/ClC-K1 repression prevents the formation of channels that facilitate the passage of chloride into the urine, thereby avoiding dysregulation in the organism’s electrolyte balance, since chloride concentration in the urine directly affects its osmolarity [30]. KLF12 is also overexpressed between 15 and 22 days after birth in the EpC of the maturing IMCD. The specificity of KLF12 expression was determined by comparing it with the expression of aquaporin 2 (AQP2), which is upregulated in the IMCD and shares similar DNA-binding sites to those of the KLFs. The co-localization of KLF12 and AQP2 suggests that KLF12 plays a key role in the positive regulation of AQP2 expression and its target gene, the urea transporter (UT-A1), by binding to a CACCC element in the UT-A1 promoter [66,67]. This is relevant because, amongst the nine aquaporins, including AQP1–8 and AQP11, that are differentially expressed along the renal tubules and collecting ducts, AQP2 is identified as one of the selective water channels [68]. This positive regulation influences the transport of water and urea into the IMCD, resulting in the accumulation of urea necessary for the proper urine concentration [69]. Figure 3 illustrates the involvement of KLFs in regulating renal organogenesis and postnatal maturation of nephrons.

KLF15 has also been associated with podocyte differentiation, since it upregulates the expression of nephrin, podocin, synaptopodin, and Wt1, all of which are essential for maintaining a differentiated phenotype and preventing the loss and detachment of podocytes [34,70]. Meanwhile, KLF4 regulates the differentiation of specific nephron segments and individual cell types by cooperating with p53 and CREB, as evidenced by the p53-CRE-KLF binding sites in the promoter regions of renal function genes AQP2, bradykinin receptor B2 (B2R) and epithelial sodium channel (ENaC) during terminal nephron differentiation [60,71].

To summarize, KLFs are vital for the proper development, structure, and function of the GFB. They safeguard ECs, facilitate the differentiation of podocytes, maintain their specialized integrity, and regulate the cell cycle and apoptosis.

### 3.2. Klf in Kidney Metabolism

Metabolic processes enable the kidneys to use, produce, and reabsorb nutrients, fulfilling their energy demands and ensuring homeostasis. To regulate these metabolic processes effectively, transcription factors and coactivators are involved in modulating the expression of genes that code for the enzymes involved. This ensures the proper occurrence of these processes.

Transcription factors such as KLFs have been closely associated with regulating metabolism, especially in the liver [72,73,74,75,76,77,78], where they regulate genes involved in lipid metabolism such as CREB, carbohydrate response element binding protein (ChREBP), sterol regulatory element binding protein 1 (SREBP-1), and peroxisome proliferator-activated receptor (PPAR). Moreover, they regulate genes involved in glycolytic metabolism, such as peroxisome proliferator-activated receptor gamma coactivator 1 alpha (PGC-1α) [72,79,80,81], because they share the same DNA binding sites in conserved CACCC sequences and GC-rich elements [82].

Variations in metabolic activity among KLFs remain unexplored; however, in vitro studies have shown that KLF6 overexpression in proximal tubule cells (PTCs) (Hk-2) exposed to high glucose concentrations increases the expression of the protein that interacts with thioredoxin (Txnip) [83]. This protein is particularly intriguing due to its involvement in glucose oxidation, which is necessary for energy acquisition. One of the most significant factors is related to the glucose transporter GLUT1. Txnip regulates this transporter and influences its localization by directly binding to it. This binding induces internalization through clathrin-coated pits [84]. Therefore, its expression suggests that peripheral glucose uptake and the utilization of this substrate could be compromised, leading to metabolic disturbances in conditions of persistently high glucose levels, such as diabetes mellitus. Thus, silencing KLF6 may represent a therapeutic target, as its inhibition significantly attenuates Txnip expression and mitigates metabolic consequences.

Within the Hk-2 cellular model, KLF14 plays an important role in lipid metabolism. The absence of KLF14 has been linked to a decrease in mitochondrial activity by reducing the expression of PPARα, since KLF14 can specifically bind to the region -222 to -209 of the PPARα promoter region and regulate its expression. This suggests an insufficient energy supply to renal tubular cells, leading to lipid accumulation and the subsequent development of tubulointerstitial fibrosis [85]. Furthermore, it inhibits the expression of carnitine palmitoyltransferase 1 (CPT1), an enzyme that facilitates the transfer of acyl groups from acyl-CoA to carnitine in the intermembrane space of the mitochondria, leading to the production of acyl-carnitine esters. By utilizing this process, the inner mitochondrial membrane allows long-chain fatty acid transportation to occur. In the next step, CPT2 transfers the acyl groups from acyl-carnitine back to CoA, ultimately restoring acyl-CoA. Subsequently, this acyl-CoA can be metabolized through a series of enzymatic reactions within the β-oxidation pathway of fatty acids [86,87].

Thus, the downregulation of PPARα causes the accumulation of lipids inside cells, which can occur in a wide range of renal cell types, including mesangial cells, podocytes, and PTCs [88]. This applies particularly to PTCs, since they rely on FAO for energy production [89]. Renal damage can occur when lipids accumulate in cells unfitted for storage. The build-up of this substance promotes lipoperoxidation, which generates ROS and leads to oxidative stress and the development of diseases like AKI and fibrosis [90]. Nevertheless, the overexpression of KLF14 counteracts these effects by enhancing mitochondrial activity, reducing lipogenesis, and decreasing lipid accumulation. In experiments conducted on living subjects, it was found that KLF15 could positively regulate both CPT1 and Acyl-CoA Acyltransferase 2 (ACAA2) by tightly binding to the PPARα binding sites [91]. The closeness of DNA suggests that KLF15 and PPARα may coordinate in governing the expression of these genes.

**Figure 4:**
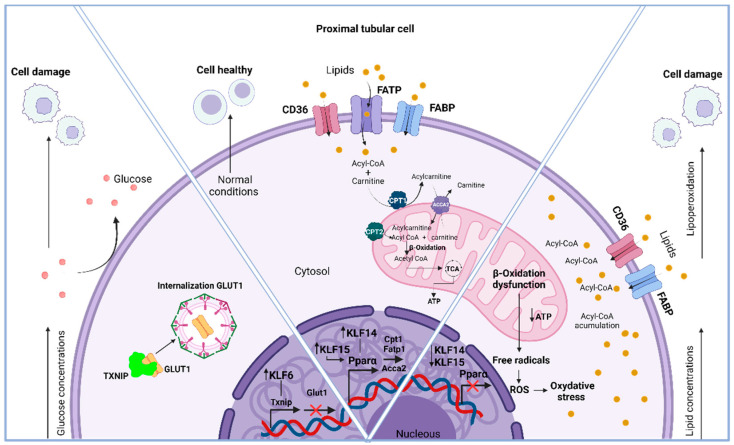
Involvement of KLFs in renal metabolism under healthy conditions and their effect in states of dysregulation.

Thus, it is crucial to have high levels of KLF14 and KLF15 to ensure sufficient energy supply, specifically to the PTCs, by utilizing lipids through FAO. Figure 4 illustrates the involvement of KLFs in renal metabolism under healthy conditions and their effect on states of dysregulation.

### 3.3. KLF in Kidney Disease

When the expression of KLFs is dysregulated, it can disrupt the various physiological processes in which they are involved. This disruption can cause poor management of inflammation, tissue repair, regeneration, and other cellular adaptations to stress.

Throughout this review, it has been noted that KLFs demonstrate distinct patterns of expression in different renal cells, greatly influencing their various functions in maintaining overall balance within the body [92]. The dysregulation of KLFs is associated with kidney diseases, including CKD, AKI, and DKD, where the pathophysiology affects the balance of glomerular, tubular, and inflammatory functions; this variability is commonly observed. Considering that specific KLFs have protective properties for the kidneys, blocking them frequently leads to the deterioration and advancement of the disease. Conversely, diseases can also be exacerbated by the excessive expression of other KLFs.

It is important to mention that AKI, which is characterized by a sudden decrease in kidney function, has the potential to progress to chronic injury and eventually develop into CKD [93]. In this context, KLF2, KLF4, KLF9, KLF10, and KLF15 have beneficial effects [94,95,96,97,98], while KLF5 promotes cell proliferation, tubular damage, and inflammation [99]. Various pathological conditions, such as diabetic nephropathy, which is characterized by albuminuria and progressive renal insufficiency [100], precipitate the development of CKD. Here, the KLFs that stand out are primarily KLF3, which induces inflammation and fibrosis, and KLF6, which facilitates EMT. Ultimately, all these diseases converge on a common outcome of renal fibrosis, characterized by excessive deposition of ECM [101,102]. The activity of each KLF in these three diseases is detailed in Table 2.

## 4. Perspective

Ongoing research is advancing our understanding of KLFs and how they affect the development of the kidneys, metabolic activity, and renal diseases. With an increasing comprehension of these factors, it is apparent that a more thorough exploration of the cellular functions in which KLFs might be involved is necessary. Thus, it is of the utmost importance to examine the specific interactions of KLFs with other transcription factors and signaling pathways. Understanding how these factors regulate gene expression in different contexts can provide valuable insights into their role in renal homeostasis and response to injury. Moreover, a detailed approach is required regarding the effects of KLFs on renal metabolism. Given that the kidneys are crucial organs for the metabolism of nutrients, such as glucose and lipids, investigating how KLFs modulate these metabolic pathways could reveal new therapeutic strategies for diseases like diabetes and CKD.

Developing experimental models that permit the study of KLF function in pathological conditions is also of great significance. The creation of in vivo and in vitro models will facilitate the identification of biomarkers and the evaluation of therapeutic interventions that could improve renal health—ultimately, incorporating cutting-edge technologies such as genetic editing, through CRISPR/Cas9, by manipulating KLF genes to study their effects on both promoting health and the development of kidney disease. Additionally, systems biology, through large data analysis with simple and accessible bioinformatics tools, can facilitate the identification of interaction networks and kidney disease biomarkers. By combining both approaches, more precise models can be developed, and therapeutic strategies can be personalized. This could open new avenues for identifying therapeutic targets and intervention strategies in kidney diseases.

In summary, studying KLFs has the potential to significantly expand our knowledge of renal development and disease. A multidisciplinary approach will be key to unlocking these mechanisms and contributing to developing new therapies that improve renal health. For this, the valuable collaboration of experts from various fields is essential. Specifically, the participation of molecular biologists and geneticists brings theoretical and practical knowledge of gene manipulation to identify its direct impact on kidney function and the regulation of homeostasis. Renal physiologists help determine the specific physiological processes of the kidneys under certain circumstances. Bioinformaticians are essential in analyzing large volumes of data, facilitating the identification of interaction networks and biomarkers associated with kidney diseases. Nephrology specialists and nutritionists, through their clinical experience and focus on nutrition and overall well-being, contribute their knowledge of the external factors influencing renal health, such as diet and lifestyle. This interdisciplinary collaboration allows for a deeper understanding of how KLFs regulate renal homeostasis, thus promoting health and helping to prevent or treat diseases.

## Figures and Tables

**Table 1:** Briefly shows the role of KLFs in nephron cells and their implication in renal physiology.

Nephron Cell Type	KLF	Role	References
Podocytes	KLF4	Induces the positive expression of E-cadherin, podocin, and nephrin by interacting with HDACs, for the maintenance of tight junctions and the SD. Induces the expression of cytokeratins (K8 and K18) that help in the cytoskeleton’s organization. Downregulates mesenchymal markers such as vimentin and α-SMA, preventing EMT and structural damage. Improves the conditions of diabetic nephropathy conditions by activating through the activation of podocyte autophagy via negative regulation of the mTOR pathway, achieved by the reduction of phosphorylated protein levels (p) mTOR and pS6K, thus preventing excessive ECM production	[34,36,51]
	KLF2	Regulates the size and distribution of transcellular pores in the ECs by inhibiting the phosphorylation of the myosin light chain. Modulates VEGF-A-mediated angiogenesis by downregulating its expression, preventing an excess of blood vessels.	[44]
Glomerular endothelial cells	KLF4	Mediates inflammation by downregulating VCAM1 induced by TNF-α, inhibiting the p65 subunit of NF-κB.	[51]
	KLF4	Attenuates the expansion of the mesangial matrix and its proliferation by negatively regulating the mTOR pathway, downregulating the expression of phosphorylated (p) mTOR and p S6K proteins, thus preventing excessive extracellular ECM production.	[51]
Mesangial cells	KLF4	Mitigates inflammation and fibrosis by decreasing the expression of pro-inflammatory cytokines, such as MCP-1, MIP-3α, and IL-8.	[55]
Proximal tubule cells	KLF11	Like KLF4, it participates in the mitigation of inflammation and fibrosis by reducing the expression of the same pro-inflammatory cytokines.	[56]
	KLF15	Decreases the expression of fibronectin by negatively regulating the MAPK pathways.	[57]

**Table 2:** The roles of KLFs on kidney disease.

Disease	KLF Group	Role	References
Chronic kidney disease	Group 1 (KLF 3, 8, and 12)	Not Available	
	Group 2 (KLF 1, 2, 4, 5, 6, and 7)	KLF2 protects endothelial cell injury through anti-inflammatory, anti-thrombotic, and anti-angiogenic effects, as it maintains the proper function of glomerular ECs. Its deficiency has been shown to lead to the progression of renal disease.	[94,103]
		KLF4 suppression causes the polarization of infiltrating macrophages into myeloid cells that accumulate in the glomerulus and tubular interstitium in CKD to shift to an M1 phenotype. The M1 phenotype of macrophages promotes the production of pro-inflammatory cytokines, such as TNFα and IL-1β. These cytokines exacerbate renal parenchymal injury and accelerate disease progression. Conversely, KLF4 expression suppresses the differentiation of infiltrating macrophages, mitigating renal damage by inhibiting TNFα expression in myeloid cells. Thus, KLF4 is considered a protective transcription factor. In addition, KLF4 mitigates inflammation and fibrosis caused by the TGF-β1-induced release of cytokines MCP-1, MIP-3α and IL-8 in human PTCs, possibly relating to the phosphorylation of KLF4 that TGF-β1 induces via SMAD and p38/MAPK signaling in VSMCs. It has even been linked to the inhibition of podocyte apoptosis by regulating the mTOR signaling pathway, which regulates cell growth, proliferation, and survival.	[55,104]
		KLF5 participates in the initiation and progression of tubulointerstitial inflammation, and its expression is increased in proliferating renal tubule cells in the cortex and medulla of fibrotic kidneys. KLF5 regulates renal fibrosis through activation of the HIF-1α-KLF5-TGF-β1 pathway, renal cell proliferation through activation of the ERK/YAP1/KLF5/cyclin D1 pathway, and tubulointerstitial inflammation with upregulation of pro-inflammatory cytokines which promotes kidney injury.	[7,28]
		KLF6 under conditions that promote renal damage and fibrosis, such as its overexpression, enables TGF-β1 to induce the loss of E-cadherin, gain in vimentin expression, and lead to EMT of PTCs. In CKD, TGF-β promotes renal fibrosis by enhancing matrix formation, cell proliferation, and cell migration via the MAPK, phosphatidylinositol 3-kinase/protein kinase B, and Smad2/3/4 pathways, subsequently elevating fibronectin, collagen, and α-SMA.	[105,106]
	Group 3 (KLF 9, 10, 11, 13, 14, and 16)	KLF11 deficiency is associated with increased renal atrophy, fibrosis, and interstitial inflammation in a mouse model of chronic renal obstruction (UUO). In KLF11 KO-UUO mice, this deficiency is linked to the upregulation of genes such as type I collagen, fibronectin, TGF-β1, IL-6, and TNF-α. These genes are associated with TGF-β signaling, fibrosis, and inflammation.	[56]
	No group (KLF 15 and 17)	KLF15 is downregulated by TGF-β1, which activates multiple intracellular signal transduction systems and MAPK pathways, including ERK and JNK, leading to renal fibrosis. Thus, KLF15 may play an anti-fibrotic factor in renal interstitial fibrosis by decreasing ECM fibronectin, type III collagen, and CTGF expression in renal fibroblast. Furthermore, the overexpression of KLF15 in mesangial cells also reduced the expression of fibronectin and type IV collagen, as well as in HEK293 cells, suggesting that the inhibition of ECM expression mediated by KLF15 may not be a cell type-specific effect.	[57,107,108]
Acute kidney injury	Group 1 (KLF 3, 8, and 12)	Not Available	
	Group 2 (KLF 1, 2, 4, 5, 6, and 7)	Overexpression of KLF4 in PTCs (HK-2) upregulates the expression of miR-101. This increase in miR-101, downregulates the expression of COL10A1, thereby suppressing EMT and renal fibrosis during the pathogenic process of renal fibrosis associated with AKI. In contrast, the inhibition of KLF4 expression, directly mediated by epigenetic regulatory enzymes, such as DNA methyltransferase 1 (Dnmt1), which hypermethylates the KLF4 promoter region, contributes to the progression of EMT in renal EpC.	[109]
		KLF5 is regulated by YAP and promotes the expression of Mst1/2, proteins involved in the Hippo signaling pathway. Activation of this pathway leads to over proliferation of tubular cells, tubular injury, and inflammation. KLF5 can be upregulated in severe AKI because of the activation of HIF-1α, which facilitates the transition to CKD. The overexpression of KLF5 promotes renal fibrosis and tubular dysfunction, exacerbating AKI. KLF6 is rapidly expressed in PTCs after AKI, contributing to the exacerbation of the disease by repressing genes involved in branched-chain amino acid catabolism, such as Bckdha, Bckdhb, Acadm, Mut, Hibch, Ivd, Mccc1, and Mccc2, which, through this metabolic process, provide intermediates necessary for the tricarboxylic acid cycle in the absence of FAO to produce ATP and meet the energy needs of the cells. In contrast, the decreased expression of KLF6 improves AKI and fibrosis through the particular preservation of the expression of the BCAA catabolic enzyme Bckdhb.	[99,110,111]
	Group 3 (KLF 9, 10, 11, 13, 14, and 16)	KLF9, which is upregulated by miR-93-5p, inhibited the expression of circHIPK3, leading to the alleviation of oxidative stress and apoptosis in an in vivo model of AKI established by ischemia/reperfusion (I/R) in C57BL/6 mice or hypoxia/reoxygenation (H/R) in HK-2 cells. The circular RNA HIPK3 (circHIPK3), derived from the HIPK3 gene, is important because of its pro-inflammatory activity.	[97]
		KLF10 is downregulated in tubular cells during AKI. This finding suggests that KLF10 protects against AKI, as its induction improves tubular regeneration through the ZBTB7A-KLF10-PTEN axis. PTEN is important because it can inhibit the PI3K/Akt pathway, which regulates cell growth, death, migration, and differentiation.	[96]
		KLF11 depletion in proximal EpC increases the expression of endothelin-1 and IL-6, leading to elevated serum creatinine and blood urea nitrogen levels, as well as damage and death of tubular capillaries in the deep cortex and outer medulla, tubular cast formation, vascular dilation, and congestion, thereby exacerbating AKI. However, KLF11 expression reduces endothelin-1-dependent renal vasoconstriction, inflammation, and aberrant renal hemodynamics.	[112]
	No group (KLF 15 and 17)	KLF15 acts as a bridge connecting the signaling of diacylglycerol kinase epsilon (DGKE) and Klotho. This DGKE/KLF15/Klotho pathway protects against renal ischemia/reperfusion injury (IRI) and AKI in a murine model. In a Xenopus laevis model, it was shown that KLF15 directly binds to enhancers and stimulates the expression of regenerative genes, including adrenoreceptor α 1A (adra1α), suggesting that KLF15 might even promote the regeneration of nephric tubules. KLF15 attenuates damage and development of glomerulosclerosis, tubulointerstitial fibrosis, and inflammation, and stabilizes the actin cytoskeleton, thereby improving renal function.	[95,113]
Diabetic kidney disease	Group 1 (KLF 3, 8, and 12)	KLF3 directly regulates the transcription of STAT3. In PTCs (HK-2) exposed to high glucose concentrations, the suppression of KLF3 mediated by miR-23a-3p resulted in the inhibition of STAT3, a protein crucial for regulating inflammation and fibrosis associated with metabolic diseases. Thus, the inhibition of KLF3 leads to a protective effect in renal disease.	[102]
	Group 2 (KLF 1, 2, 4, 5, 6, and 7)	KLF2 is upregulated by insulin treatment and downregulated by high glucose concentrations in cultured ECs from diabetic mice. Even in a KLF2 KO ± EC model, it was determined that reduced KLF2 expression induced more endothelial cell damage. Additionally, in glomerular endothelial cell (GEC)-specific KLF2 knockout mice with streptozotocin-induced diabetes, it was found that the expression of podocyte-specific genes encoding nephrin, podocin, and synaptopodin in the kidney was decreased compared to wild-type diabetic mice. This suggests the possibility of an interaction mechanism between GECs and podocytes mediated by KLF2. The deletion of KLF2 (knockout, KO) in the glomeruli reduces the expression of several of its target genes, including endothelial nitric oxide synthase (eNOS), zonula occludens-1 (ZO-1), the glycocalyx, fms-related tyrosine kinase 1 (Flt1), tyrosine kinase with immunoglobulin-like and EGF-like domains 2 (Tie2), and angiopoietin 1 (Angpt1). These genes are primarily involved in the function and integrity of the vascular endothelium, which is why KLF2 is considered a vasoprotective factor. A potential mechanism by which KLF2 may decrease its expression under high glucose conditions has been demonstrated in human umbilical vein ECs, where KLF2 transcriptional silencing is mediated by FOXO1, which binds to the KLF2 promoter region.	[114,115,116]
		KLF4 overexpression induces podocyte autophagy, protecting the tissue from damage in DKD. Furthermore, it suppresses cell proliferation and differentiation during fibrosis and inhibits EMT processes. Hyperglycemia also decreases KLF4 expression and increases TGF-β expression leading to unregulated inflammation in renal tissue.	[51,114,115]
		KLF5 is overexpressed in the collecting duct EpC found in diabetic kidney and tubulointerstitial disease and it is associated with alterations like an expansion of mesangial matrix and tubular interstitial space, podocyte damage, and glomerular basement membrane thickening, showing that KLF5 plays a pivotal role in the initiation and progression of renal inflammation. The inverse expression of KLF4 and KLF5 in the pathogenesis of renal fibrosis is modulated by a matrix stiffness-regulated extracellular signal-regulated kinase (ERK), which increases the protein level and nuclear translocation of mechanosensitive YAP1, preventing the degradation of KLF5. KLF5 is upregulated under hyperglycemic conditions through lactylation of lysine 14 on histone H3 (H3K14la). KLF5 binds to the promoter of the gene encoding E-cadherin (Cadherin 1, cdh1) and inhibits its transcription, promoting disease progression. This lactylation results from lactate accumulation because of the metabolic reprogramming that renal PTCs undergo in a hyperglycemic state, specifically the shift from OXPHOS to glycolysis.	[10,28,117]
		KLF6 overexpression, under conditions that promote renal damage and fibrosis such as diabetic nephropathy, enables TGF-β1 to induce the loss of E-cadherin, gain in vimentin expression, and EMT of PTCs. In CKD, TGF-β promotes renal fibrosis by enhancing matrix formation, cell proliferation, and cell migration via MAPK, phosphatidylinositol 3-kinase/protein kinase B, and Smad2/3/4 pathways, subsequently elevating fibronectin, collagen, and α-SMA.	[106]
	Group 3 (KLF 9, 10, 11, 13, 14, and 16)	KLF10 Activates KDM6A and induces proteinuria, kidney damage, and fibrosis under diabetic conditions. It represses nephrin, WT1, podocin, and synaptophysin in podocytes, and increases expression of type I and III collagen, fibronectin, and metalloproteinases.	[114,118,119]
	No group (KLF15 and KLF17)	KLF15 modulates mitochondrial biogenesis and homeostasis through the SIRT1-PGC-1α pathway in mouse mesangial cells associated with diabetic nephropathy. This finding was determined through enrichment analysis, which identifies KLF15 as a therapeutic target.	[120]
